# Characterization of the *SWEET* Gene Family in Longan (*Dimocarpus longan*) and the Role of *DlSWEET1* in Cold Tolerance

**DOI:** 10.3390/ijms23168914

**Published:** 2022-08-10

**Authors:** Ting Fang, Ya Rao, Mengzhen Wang, Yun Li, Yujun Liu, Pengpeng Xiong, Lihui Zeng

**Affiliations:** Institute of Genetics and Breeding in Horticultural Plants, College of Horticulture, Fujian Agriculture and Forestry University, Fuzhou 350002, China

**Keywords:** *Dimocarpus longan*, SWEET, sugar accumulation, cold tolerance

## Abstract

Sugars will eventually be exported transporters (SWEET), a group of relatively novel sugar transporters, that play important roles in phloem loading, seed and fruit development, pollen development, and stress response in plants. Longan (*Dimocarpus longan*), a subtropic fruit tree with high economic value, is sensitive to cold. However, whether the *SWEET* gene family plays a role in conferring cold tolerance upon longan remains unknown. Here, a total of 20 longan SWEET (*DlSWEET*) genes were identified, and their phylogenetic relationships, gene structures, *cis*-acting elements, and tissue-specific expression patterns were systematically analyzed. This family is divided into four clades. Gene structures and motifs analyses indicated that the majority of *DlSWEETs* in each clade shared similar exon–intron organization and conserved motifs. Tissue-specific gene expression suggested diverse possible functions for *DlSWEET* genes. *Cis*-elements analysis and quantitative real-time PCR (qRT-PCR) analysis revealed that *DlSWEET1* responded to cold stress. Notably, the overexpression of *DlSWEET1* improved cold tolerance in transgenic *Arabidopsis*, suggesting that *DlSWEET1* might play a positive role in *D. longan’s* responses to cold stress. Together, these results contribute to a better understanding of SWEET genes, which could serve as a foundation for the further functional identification of these genes.

## 1. Introduction

Sugars are not only the predominant carbon and energy sources for both eukaryotes and prokaryotes, but also the essential signaling molecules for diverse physiological processes [1]. In addition, sugars are the main determiners of fruit quality and flavor [2,3]. In plants, sugars are the main products of photosynthesis, which occurs mainly in the stromal cells of the chloroplast, and are translocated to the sink organs via long-distance transport [4,5]. However, the movement of sugar between tissues via the phloem requires the assistance of sugar transporters [6,7]. To date, various sugar transporters have been identified in plants and they can be grouped into three families: monosaccharide transporter-like (MST), sucrose transporters (SUTs/SUCs), and sugars will eventually be exported transporters (SWEETs) [8].

The SWEET families are a recently discovered protein family of carbohydrate transporters in plants and mammals [9], which feature the MtN3/Saliva motif (PF03083) and seven transmembrane helices (TMHs) [10]. The eukaryotic SWEETs have a 3-1-3 TMH structure, which is organized as tandem repeats of two 3-TMH domains separated by a single transmembrane domain [9,11,12,13]. On the contrary, prokaryotic SWEETs (designated as SemiSWEETs) contain only a single triple helix bundle (THB), indicating that SemiSWEETs might have evolved from the duplication of the THB [10,14,15].

To date, the genome-wide analysis of *SWEET* genes has been comprehensively conducted in several plant species, such as *A. thaliana* [9], rice (*Oryza sativa*) [13], lychee (*Litchi chinensis* Sonn) [16], and pomegranate (*Punica granatum*) [17]. Biochemical and functional analyses have shown that *SWEET* genes are involved in many different functions, such as phloem loading [9], nectar secretion [18], pollen development [19], modulating gibberellins response [20], senescence [21], abiotic stress response [22,23,24,25,26,27], host–pathogen interactions [28,29,30,31], and seed and fruit development [32,33,34].

Longan (*Dimocarpus longan* L.), which belongs to the family of Sapindaceae, is an economically important evergreen fruit crop [35]. China is the largest producer of longan and account for 70% and more than 50% of the world’s acreage and production, respectively [36]. Sugar composition and content in the aril of longan fruit is an important factor determining the quality of longan fruit. Consequently, high sweetness has become a major objective for longan breeding. Cold stress is a major environmental factor that adversely affects plant growth and development, as well as the product quality and yield. Longan, which originated in South China or Southeast Asia, is sensitive to cold, although longan is widely cultivated in the tropical and subtropical regions of the world [37]. In recent years, unpredictable frost-inducing weather has occurred in southern China, resulting in severe economic losses in the longan industry; thus, a better understanding of the molecular response mechanisms of longan cold stress is urgent.

In the present study, 20 *SWEET* genes in longan (termed *DlSWEET*) were identified, and their phylogenetic relationship, gene structures, protein motifs, *cis*-acting elements, and tissue-specific expression patterns were analyzed. We also determined the expression patterns of some selected *DlSWEET* genes during fruit development stages and in response to different abiotic stress treatments. Additionally, the *DlSWEET1* gene was introduced into *A. thaliana* by *Agrobacterium*-mediated method to investigate its function. The results will serve to facilitate our understanding of the function of SWEET genes.

## 2. Results

### 2.1. Identification and Phylogenetic Analysis of SWEET Proteins in Longan

A total of 20 *SWEET* genes were identified and renamed from *DlSWEET1* to *DlSWEET16b* according to their phylogenetic relationship with 17 *AtSWEET* (Table 1). Gene characteristics, including gene size, coding sequence size, protein length, isoelectric point (pI), molecular weight (MW), and grand average of hydropathicity (GRAVY) value were analyzed. The predicted size of 20 DlSWEET proteins ranged from 140 (DlSWEET16b, 15.48 KDa) to 440 (DlSWEET2b, 49.29 KDa) amino acids, and the predicted isoelectric points ranged from 5.77 (DlSWEET15) to 9.54 (DlSWEET4). The predicted GRAVY value of DlSWEET proteins ranged from 0.236 to 0.914, indicating hydrophobic properties.

**Figure 1:**
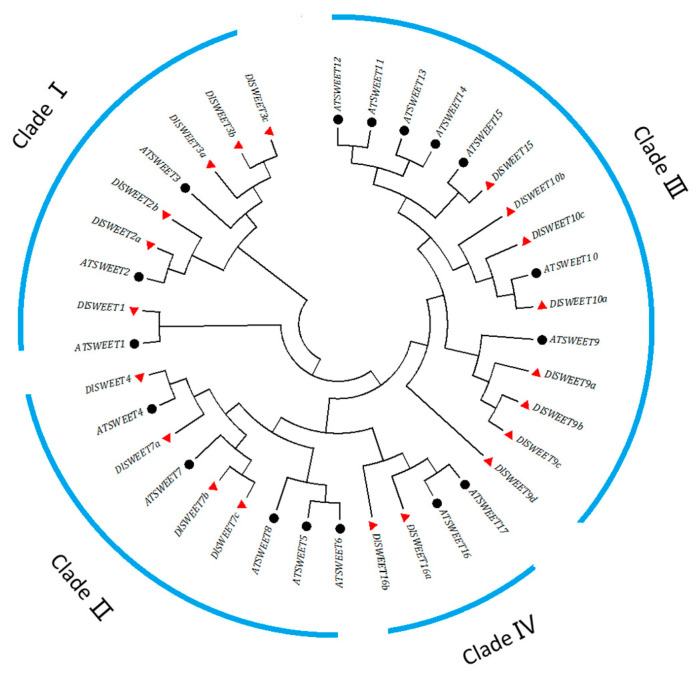
The phylogenetic analysis of SWEET family genes of *Arabidopsis* and *D. longan*. At, *A. thaliana*; Dl, *D. longan*. AtSWEET and DlSWEET proteins are identified by red triangles and black dot, respectively.

The evolutionary relationships of DlSWEET family members were assessed by constructing an NJ phylogenetic tree (Figure 1). The results showed that DlSWEET proteins were clearly grouped into four different clades. Clade III is the largest groups, which contained eight *DlSWEETs* (*DlSWEET9a*/*9b*/*9c*/*9d*/*10a*/*10b*/*10c*/*15*), while clade IV had only two members (*DlSWEET16a*/*16b*). Clades I and II contained six and four members, respectively.

### 2.2. Analysis of Transmembrane Domains and Conserved Motifs

To further investigate the features of DlSWEET proteins, TMHMM Server v2.0 (http://www.cbs.dtu.dk/services/TMHMM-2.0/) (Copenhagen, Danmark) was used to predict the transmembrane domains (Table 1 and Figure S1). The results showed that only ten DlSWEET proteins contain seven TMHs, and five DlSWEET proteins had six TMHs. In addition, four DlSWEET proteins had five TMHs. Interestingly, DlSWEET16b contained three TMHs, which is observed in eukaryotes.

**Figure 2:**
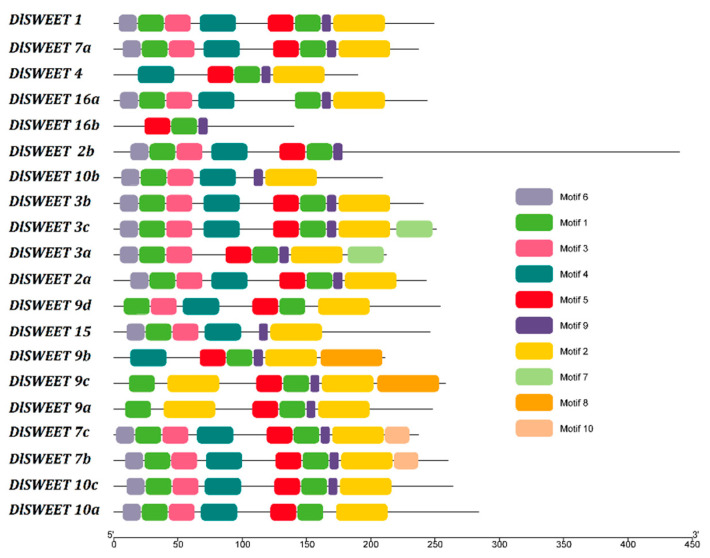
Conserved motif analysis of DlSWEET proteins.

The conserved motifs of the DlSWEET proteins were examined using MEME program in DlSWEET proteins (Figure 2 and Figure S2 and Table S1). The result showed that 10 conserved motifs were identified among the 20 DlSWEETs. Motifs 1 and 2 were observed in 20 DlSWEETs, while motif 7 and motif 10 only appeared in two DlSWEETs each.

### 2.3. Exon–Intro Organization of DlSWEET Genes

**Figure 3:**
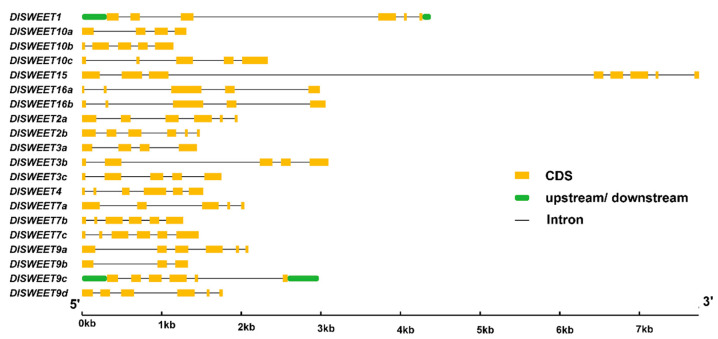
The gene structures of the *DlSWEET* genes, include introns (black lines), exons (CDSs, yellow rectangles), and untranslated regions (UTRs, green round-corner rectangles).

To gain insights into the structure of *DlSWEET* genes, the introns and exons organization, which play important roles in the evolution of multiple gene families, were analyzed (Figure 3). The results showed that all *DlSWEET* genes contained introns, ranging from two to seven. Furthermore, nine *DlSWEET* genes (*DlSWEET1/2a/3a/3b/3c/7a/10a/10c/16a*) contained six exons, while seven *DlSWEET* genes (*DlSWEET7b/7c/9a/9c/9d/10b/15*) harbored five exons, two genes (*DlSWEET4/9b*) displayed four exons, and *DlSWEET2b* and *DlSWEET16b* possessed eight and three exons, respectively. Although some similar *DlSWEET* genes in the same clade shared a similar gene structure, for example, *DlSWEET3a/3b/3c* contains 6 exons and 5 introns, differences also existed in their length due to the introns.

### 2.4. Cis-Acting Elements in the Promoters of DlSWEETs

**Figure 4:**
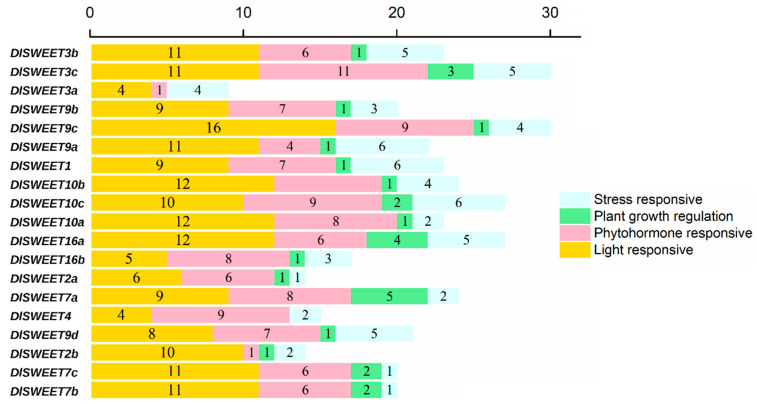
*Cis*-acting regulatory elements in *DlSWEETs* promoters.

The regulatory role of *DlSWEETs* was studied by gathering the 2000 bp upstream regions of 19 *DlSWEETs* (*DlSWEET15* is located from 142 to 2474 bp in scaffold601, so we could not obtain the promoter sequence from the longan genome), and the transcriptional response elements of *DlSWEETs* were predicted using the PLACE database (Figure 4 and Table S2). A series of *cis*-elements involved in plant growth regulation processes were identified, such as GCN4 motifs found in the promoters of four genes (*DlSWEET2b/9b/9c/16a*). Additionally, 20 light-responsive *cis*-elements were identified; the BOX 4 element, in particular, existed in all promoters of *DlSWEETs* genes, suggesting that the light may play important roles in regulating the expression of *DlSWEETs* genes. Notably, many plant hormones and stress-responsive *cis*-elements, such as ABRE, AuxRR-core, LTR, and MBS were present in the promoter region of *DlSWEETs*, suggesting that some *DlSWEET* genes may responsed to plant hormone and stress treatments.

### 2.5. Tissue-Specific Expression Patterns of DlSWEET Genes

**Figure 5:**
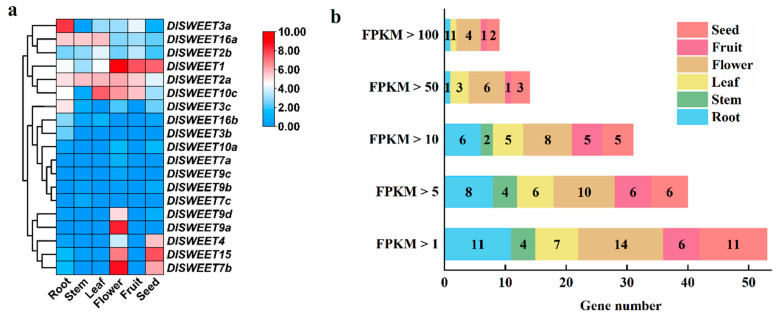
Tissue-specific expression profiles of *DlSWEET* genes. (**a**) Heatmap of expression levels for *DlSWEET* genes in six tissues (root, stem, leaf, flower, fruit and seed). The color scale represents log2 (FPKM+1) normalized transformed counts where blue indicates low expression and red indicates high expression. (**b**) The sum of the *DlSWEET* genes with different transcriptional abundance.

To understand the tissue-specific-expression patterns of *DlSWEET* genes, transcripts abundance of *DlSWEET* genes in different tissues, including root, stem, leaf, flower, fruit, and seed, were analyzed based on publicly available RNA-seq datasets (GSE84467) [38] (Figure 5). As is shown in Figure 5a, the *SWEET* genes showed different expression patterns in different longan tissues (*DlSWEET10b* had no record in the datasets). Among the 19 *DlSWEETs*, 16 were expressed at relatively high levels (FPKM value > 1) in at least one tissue, including 11, 4, 7, 14, 6, and 11 *DlSWEETs* in root, stem, leaf, flower, fruit, and seed, respectively (Figure 5b). Interestingly, the FPKM values of some *DlSWEETs* were higher than 50, demonstrating that they may be important for longan development. For instance, three genes (*DlSWEET2a/10c/16a*) and six genes (*DlSWEET1/2a/7b/9a/10c/15*) showed a higher expression level in leaf and flower, respectively, indicating their potential roles in regulating longan leaf and flower development. Notably, *DlSWEET1* showed higher expression levels in flower, fruit and seeds, indicating that it may play an important role in longan growth and development.

### 2.6. Soluble Sugar and Expression Patterns of DlSWEET Genes during Fruit Development

**Figure 6:**
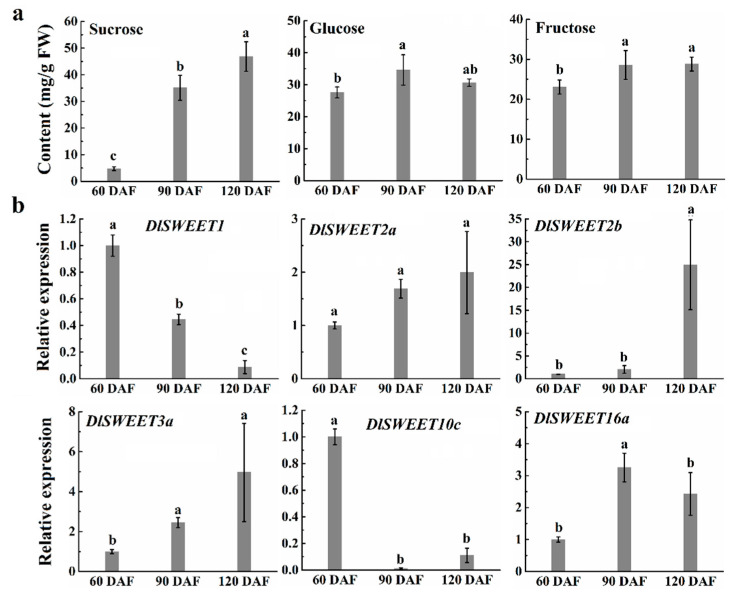
Soluble sugars content and expression profiles of *DlSWEET* genes during longan fruit development. (**a**) Content of sucrose, fructose, and glucose during “Qingkebaoyuan” fruit development. (**b**) Expression analysis of six *DlSWEET* genes during “Qingkebaoyuan” fruit development by qRT-PCR analysis. DAF, days after flowering. Values are presented as mean ± standard error (SE) (*n* = 3). Means denoted by the same letter are not significantly different at *p* < 0.05.

As is shown in Figure 6a, the sucrose content increased rapidly from 60 DAF to 120 DAF, with concentrations ranging from 4.72 to 46.81 g·kg^−1^ fresh weight (FW). The concentrations of fructose exhibited a slightly increasing trend as the fruit developed. Additionally, the glucose content increased slightly from 60 DAF to 90 DAF and then slightly decreased at the later stage of ripening. Overall, sucrose is the main component of soluble sugars in mature longan fruit. Considering the potential roles of *DlSWEETs* in the soluble sugar accumulation, we investigated the transcript abundance of six genes during longan fruit development using the qRT-PCR method (Figure 6b). Two genes (*DlSWEET1/10c*) contained extremely high expression levels in fruit at 60 DAF, but showed a decrease in transcript abundance throughout fruit development. On the contrary, the other four genes (*DlSWEET2a/2b/3a/16a*) showed relatively low expression levels at 60 DAF and then increased during fruit development, which are closely connected to the change in sugar content during longan fruit development.

### 2.7. Expression Patterns of DlSWEET Genes under Abiotic Stress Condition

**Figure 7:**
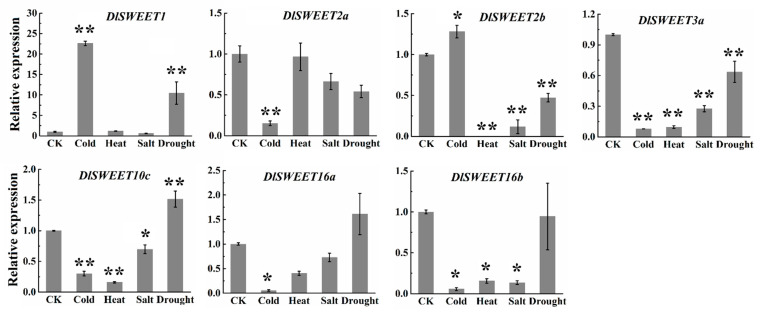
Expression analysis of the seven *DlSWEET* genes under various abiotic stress. Values presented as mean ± standard error (SE) (*n* = 3). Small star above the bars indicate significant differences (* *p* ≤ 0.05, ** *p* ≤ 0.01).

To detect whether the *DlSWEET* genes were induced by different abiotic stresses, qRT-PCR was performed to determine the expression levels of the seven *DlSWEET* genes responding to cold, heat, salt, and drought treatments in seedlings (Figure 7). Overall, the levels of expression of the seven *DlSWEET* genes differed among the four stress treatments. In detail, all the genes’s expression levels were significantly changed by cold stresses, and only *DlSWEET1* was up-regulated by cold stress, while the other genes were down-regulated by cold, indicating that *DlSWEET1* may play an important role in longan cold response. Four genes (*DlSWEET2b/3a/10c/16b*) were down-regulated by heat and salt stresses, respectively; two genes (*DlSWEET2b/3a*) were down-regulated by drought stress, whereas two genes (*DlSWEET1/10c*) were up-regulated by drought stress.

### 2.8. Overexpression of DlSWEET1 Enhances Cold Tolerance in Transgenic Arabidopsis Plants

**Figure 8:**
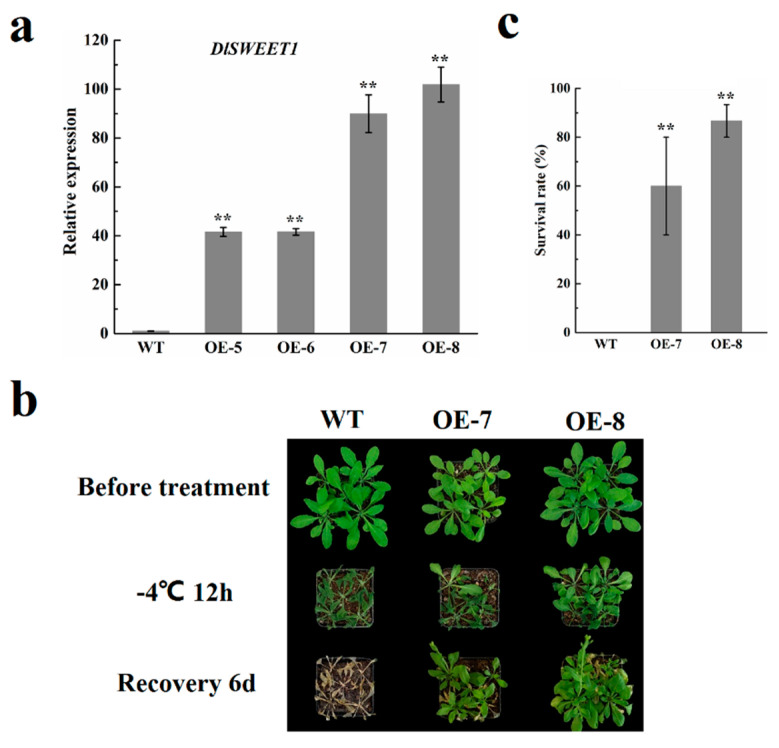
Overexpression of *DlSWEET1* in *Arabidopsis* improved cold tolerance. (**a**) The relative expression levels of *DlSWEET1* in WT and T3 transgenic *Arabidopsis* lines. *AtACTIN2* was used as an internal control. (**b**) Phenotypes of *DlSWEET1* transgenic *Arabidopsis* lines (OE-7 and OE-8) and WT under low-temperature stress and recovery. (**c**) Survival rates of the transgenic lines and WT plants after freezing. Values presented as mean ± standard error (SE) (*n* = 3). Small star above the bars indicate significant differences (** *p* ≤ 0.01).

To further explore the roles of *DlSWEET1* in cold tolerance in plants, the *35S: DlSWEET1: pSAK277* construct was transferred into *A. thaliana*, and four independent transgenic lines (T1 generation) were obtained based on kanamycin resistance selection and genomic PCR verification. T3 homozygous transgenic lines were used for cold tolerance assessments. Among the T3 generation transformed lines, four lines (OE-5, OE-6, OE-7, and OE-8) presented high expression of *DlSWEET1* by qRT-PCR with a wild-type (WT) *Arabidopsis* line as a negative control (Figure 8a). Meanwhile, we further observed the frost damage to leaves of transgenic plants (OE-7 and OE-8) and WT plants. As is shown in Figure 8b, there was no apparent difference in phenotype between the transgenic and WT plants. When they were treated at −4 °C for 12 h, after which they were allowed to recover for 6 days at 25 °C, all WT plants died, while most transgenic plants stayed alive and resumed growing (Figure 8b). The survival rate of transgenic *Arabidopsis* lines was significantly higher than the WT line at 60.00% for OE-7 and 86.67% for OE-8, compared to 0% for WT (Figure 8c). These results indicated that the overexpression of *DlSWEET1* could improve the freezing resistance of plants.

## 3. Discussion

SWEET proteins play key roles in plant growth and development by regulating sugar translocation and allocation [39,40]. To date, genome-wide analyses have identified a variable number of *SWEET* genes in over 20 plant species, and the number of reported *SWEET* gene members varies from 7 to 108, which may be caused by gene duplication, including tandem or segmental duplication [41,42,43]. In this study, 20 *SWEET* genes were identified in longan, similar to in *Arabidopsis* [9], rice [13], lychee [16], and grape [44]. These genes were classified into four clades according to their phylogenetic evolutionary relationship (Figure 2), which was consistent with previous studies [45,46,47,48]. Notably, a gene structure analysis indicated that nine *DlSWEET* genes contained six exons. Similar results were also observed in soybean [41], tomato [49], pear [50], and cucumber [51], suggesting that *SWEET* gene members are highly conserved during evolution.

Plant SWEET proteins play diverse physiological functions and can transport different sugar molecules [5,10,13]. Based on tissue-specific expression patterns of *DlSWEET* genes, *DlSWEET9a* was highly expressed in flowers (Figure 5), and its homologous gene was found to be involved in nectar secretion in *Arabidopsis*, *Brassica,* and *Nicotiana* [18]. The results indicated that *DlSWEET9a* appeared to play a key role in longan nectar secretion. The stem participates in many physiological and biochemical processes in plants. Sugar is an essential organic substance in the growth and development of the stem. *DlSWEET16a* was expressed highly in the stem (Figure 5), and the same result was observed in its homologous gene (*AT-SWEET16-1*) in *Annona squamosa* L. [52], suggesting that *DlSWEET16a* may be involved in stem growth and development. *DlSWEET15* had higher expression levels in flowers and seeds than other tissues, suggesting its function in specialized organs. In *Arabidopsis*, *AtSWEET15* was expressed in both the seed coat and endosperm, and acts as a sucrose transporter in seed coat efflux and seed filling [53]. These results suggest that *DlSWEET15* may participate in longan seed development.

Sugar content is an important factor for determining fruits’ organoleptic quality [2]. As is shown in Figure 6, the content of soluble sugars increased during fruit development and sucrose was the dominating soluble sugar in mature longan fruit. Although 20 *SWEET* gene members were identified in longan, we only detected the transcripts of six *DlSWEETs* in longan fruit. Among these six *DlSWEETs*, four genes (*DlSWEET1/2a/2b/3a*) belong to clade I, in which SWEET proteins mainly transport monosaccharides [9]. In addition, the expression level of *DlSWEET1* declined during fruit development, but the transcripts level of the other three genes increased, suggesting that *DlSWEET1* may play a role in transporting monosaccharides in the early stages of fruit development, while *DlSWEET2a/2b/3a* comes to play the roles at the fruit expansion and mature stages. In clade III, *SWEETs* in *Arabidopsis* and rice transport disaccharides, mainly sucrose [9,12]. Although *DlSWEET10c* belongs to clade III, its transcript abundance is inconsistent with the sucrose content during fruit development. The cause of the discrepancy may be a rapid conversion of sucrose to glucose or fructose at the early fruit development stage.

Longan is frequently challenged by abiotic stressors, for instance, extreme temperatures, drought, and high salinity. Sugars accumulate in plant cells to reduce the osmotic potential, which is conducive to the normal growth of plants under abiotic stress conditions [54,55]. Previous reports have shown that the *SWEET* genes were involved in abiotic stress [25,26,49]. The results showed that seven genes showed significantly up- or down-regulated expression in at least one tested treatment (Figure 7). Under drought conditions, two genes’ (*DlSWEET1/10c*) levels were significantly increased. Similar findings were observed in cabbage [31]. Although the seven genes were significantly induced by cold stress, only *DlSWEET1* showed a significantly up-regulated trend. The same result was also observed in tea, in which the expression level of *CsSWEET1* increased markedly during cold acclimation [56]. *Cis*-elements analysis showed that a TC-rich repeats element, which is involved in defense and stress responsiveness, existed in the promoter of *DlSWEET1*, indicating that *DlSWEET1* may play an important role in the longan cold response. Moreover, the overexpression of *DlSWEET1* conferred its tolerance to cold stress in transgenic *Arabidopsis*. However, due to the difference between *Arabidopsis* and longan, the function of DlSWEET1 in longan cold-response warrants further investigation.

## 4. Materials and Methods

### 4.1. Plant Materials, Stress Treatments, and Measurement of Soluble Sugars Content

To investigate the function of DlSWEETs involved in longan fruit sugar accumulation, we used fruits of Longan (*Dimocarpus longan* L.) cultivar “Qingkebaoyuan”. The trees were grown in the Fruit Research Institute, Fujian Academy of Agricultural Sciences, Fuzhou, China. The fruit samples were randomly picked from three trees, under standard cultivation conditions, at 60, 90, and 120 days after flowering (DAF). Soluble sugars in longan pulp were extracted and measured according to the previously reported protocol [57].

“Honghezi” seedlings were grown for six month in Fujian Agriculture and Forestry University, Fuzhou, China, which were used for abiotic stress treatments. For heat and cold stresses, seedlings were grown at 40 °C or 4 °C for 4 h, respectively. For salt and drought stresses, seedlings were treated with NaCl (200 mM) or PEG6000 (20%) for 4 h at 28 °C, respectively. Each treatment had three replicates of three plants. In addition, three seedlings grown at 28 °C were used as a control. After treatment, the leaf samples of the same leaf position were collected and immediately frozen in liquid nitrogen and stored at −80 °C for further analysis.

### 4.2. Identification, Phylogenetic Analysis, and Motifs Prediction of SWEET Protein in Longan

Systematic BLAST homology searches using amino sequences of 17 *Arabidopsis* SWEETs proteins [9] were performed on the longan genome (BLASTP, E-value ≤ 1 × 10^−5^) (http://gigadb.org/dataset/100276) (accessed on 26 March 2019) [38]. A phylogenetic tree was constructed with 17 AtSWEET and 20 DlSWEET protein sequences using MEGA6 by employing the neighbor-joining (NJ) method with a bootstrap value of 1000 [58].

The MEME (Multiple EM for Motif Elicitation) program (http://meme.nbcr.net/meme/cgi-bin/meme.cgi) (accessed on 31 March 2019) was used to analyze the motifs of DlSWEET proteins, with the parameters set to the following: maximum number of motifs 600, motif width 15–60, and number 15 [59].

### 4.3. Cis-Elements Search and Exon–Intron Organization of DlSWEET Genes

The exon–intron organization of the DlSWEETs were analyzed and visualized in GSDS 2.0 (gene structure display server) [60]. Furthermore, the 2000 bp promoter regions of DlSWEET were analyzed in PLACE (https://www.dna.affrc.go.jp/PLACE/?action=newplace) (accessed on 1 April 2019), and the distribution of four categories of *cis*-acting elements was visualized in the TBtools toolkit [61].

### 4.4. Expression Pattern Analysis of DlSWEET Genes

To gain insights into the *DlSWEET* genes expression patterns, we utilized the transcriptome data, which included different longan tissues (https://www.ncbi.nlm.nih.gov/geo/query/acc.cgi?acc=GSE84467) (accessed on 31 March 2019) [38]. The gene expression values were normalized by fragments per kilobase million (FPKM) and log2-transformed. The heat maps were plotted using the software TBtools [61].

### 4.5. RNA Isolation and qRT-PCR Analysis

Total RNA was extracted using an RNAprep Pure Plant Kit (TIANGEN, Beijing, China) with three biological replicates. Then, approximately 1 µg of total RNA per sample was used as the template for reverse-transcription via TransScript One-Step gDNA Removal and cDNA Synthesis SuperMix (TRANS, Beijing, China). The RT-qPCR was performed using SYBR Green I Master Mix (Takara, Dalian, China) and a LightCycler 96 Real-Time PCR Systems (Roche, Basel, Switzerland). The relative expression levels were calculated with the formula 2^−ΔΔCT^ method with three biological and three technical replicates [62]. All primer sequences used in this study are listed in Table S3.

### 4.6. Functional Analysis of DlSWEET1-Overexpressing Transgenic Arabidopsis Thaliana

The full-length cDNA sequence of *DlSWEET1* was cloned into the pSAK277 vector. The *35S: DlSWEET1* plasmid was transferred into Agrobacterium strain GV3101 by the freeze–thaw method, and transferred into the *Arabidopsis* plants using the floral dip method. The *Arabidopsis* ecotype Columbia-0 (col-0) was used as the wild type (WT) in this study. Transformed plants were selected on the basis of their resistance to kanamycin, and 3-weeks-old T3 homozygous plants and WT seedlings were used for further experiments. Two transgenic lines and WT seedlings were transferred to 4 °C growth chambers with the same ambient conditions for 48 h and then were exposed to −4 °C for 12 h, followed by 12 h of darkness at 4 °C, after which they were returned to normal conditions for recovery for 6 days. The plant survival rates and phenotypes were recorded.

### 4.7. Statistical Analyses

All reported values are presented as mean ± standard error (SE, *n* = 3). The means are compared using the Student’s *t*-test at the 0.05 and 0.01 levels of significance.

## 5. Conclusions

In summary, 20 longan SWEET genes (*DlSWEETs*) were identified in the *D. longan* genome. A phylogenetic analysis showed that *DlSWEETs* were grouped into four different subfamilies. Tissue-specific expression patterns analysis showed that *DlSWEET* genes may play various roles in the development of longan tissues. qRT-PCR analyses implied that three and four *DlSWEET* genes might be involved in abiotic stress response and fruit sugar accumulation, respectively. Furthermore, the overexpression of the *DlSWEET1* gene improved cold stress tolerance in transgenic *Arabidopsis*. This work will contribute to the follow-up study of the functional characteristics of *DlSWEET* genes and the cultivation of high-quality *D. longan* varieties. 

## Figures and Tables

**Table 1:** Characteristics of 20 *DlSWEET* genes.

Gene ID	Gene Name	Clade	Location	gDNA (bp)	CDS (bp)	Protein (aa)	PI	MV (KDa)	GRAVY	TMHs
Dlo_004842.1	*DlSWEET1*	I	scaffold132:1222727..1224250(+)	1524	750	249	9.44	27.20	0.678	7
Dlo_011035.1	*DlSWEET2a*	I	scaffold208:248691..251666(−)	2976	732	243	9.46	27.10	0.77	7
Dlo_031144.1	*DlSWEET2b*	I	scaffold81:587713..595463(−)	7751	1323	440	9.32	49.29	0.236	6
Dlo_001364.1	*DlSWEET3a*	I	scaffold105:240059..241538(−)	1480	639	212	9.24	24.07	0.509	6
Dlo_001358.3	*DlSWEET3b*	I	scaffold105:129309..133690(−)	4382	726	241	8.54	26.49	0.559	7
Dlo_001362.1	*DlSWEET3c*	I	scaffold105:227382..229337(−)	1956	756	251	9.06	28.19	0.459	7
Dlo_016654.1	*DlSWEET4*	II	scaffold32:314489..315798(−)	1310	573	190	9.54	21.48	0.729	5
Dlo_012330.1	*DlSWEET7a*	II	scaffold23:111069..112837(−)	1769	714	237	9.28	26.51	0.833	7
Dlo_036665.1	*DlSWEET7b*	II	scaffold1269:54809..57866(+)	3058	780	260	9.1	29.08	0.767	7
Dlo_035889.1	*DlSWEET7c*	II	scaffold1269:54830..57818(+)	2989	711	237	9	26.66	0.914	6
Dlo_002779.1	*DlSWEET9a*	III	scaffold1141:110734..112484(+)	1751	747	248	9.38	27.76	0.496	7
Dlo_002777.1	*DlSWEET9b*	III	scaffold1141:86225..87669(+)	1445	636	211	9.15	23.77	0.602	5
Dlo_002778.1	*DlSWEET9c*	III	scaffold1141:97643..100738(+)	3096	777	258	9.28	28.98	0.416	7
Dlo_024131.1	*DlSWEET9d*	III	scaffold539:83282..84431(+)	1150	765	254	9.01	28.42	0.531	6
Dlo_006037.1	*DlSWEET10a*	III	scaffold1436:78359..79825(+)	1467	855	284	9.4	32.10	0.603	7
Dlo_005388.1	*DlSWEET10b*	III	scaffold139:126837..128875(−)	2039	630	209	8.4	23.76	0.482	5
Dlo_006035.1	*DlSWEET10c*	III	scaffold1436:44684..45953(+)	1270	810	269	9.17	29.76	0.645	7
Dlo_026392.1	*DlSWEET15*	III	scaffold601:142..2474(+)	2333	741	246	5.77	27.80	0.574	5
Dlo_006477.1	*DlSWEET16a*	IV	scaffold148:330655..332743(−)	2089	735	244	8.56	27.11	0.645	6
Dlo_006478.1	*DlSWEET16b*	IV	scaffold148:336807..338137(−)	1331	423	140	6.17	15.48	0.344	3

## Data Availability

Not applicable.

## Supplementary Materials

The supporting information can be downloaded at: https://www.mdpi.com/article/10.3390/ijms23168914/s1.
