# Supplementary figures and images for: Characterization of the SWEET Gene Family in Longan (Dimocarpus longan) and the Role of DlSWEET1 in Cold Tolerance
Source: Int J Mol Sci. 2022 Aug 10;23(16):8914. doi: 10.3390/ijms23168914 (PMC9408694; doi:10.3390/ijms23168914)

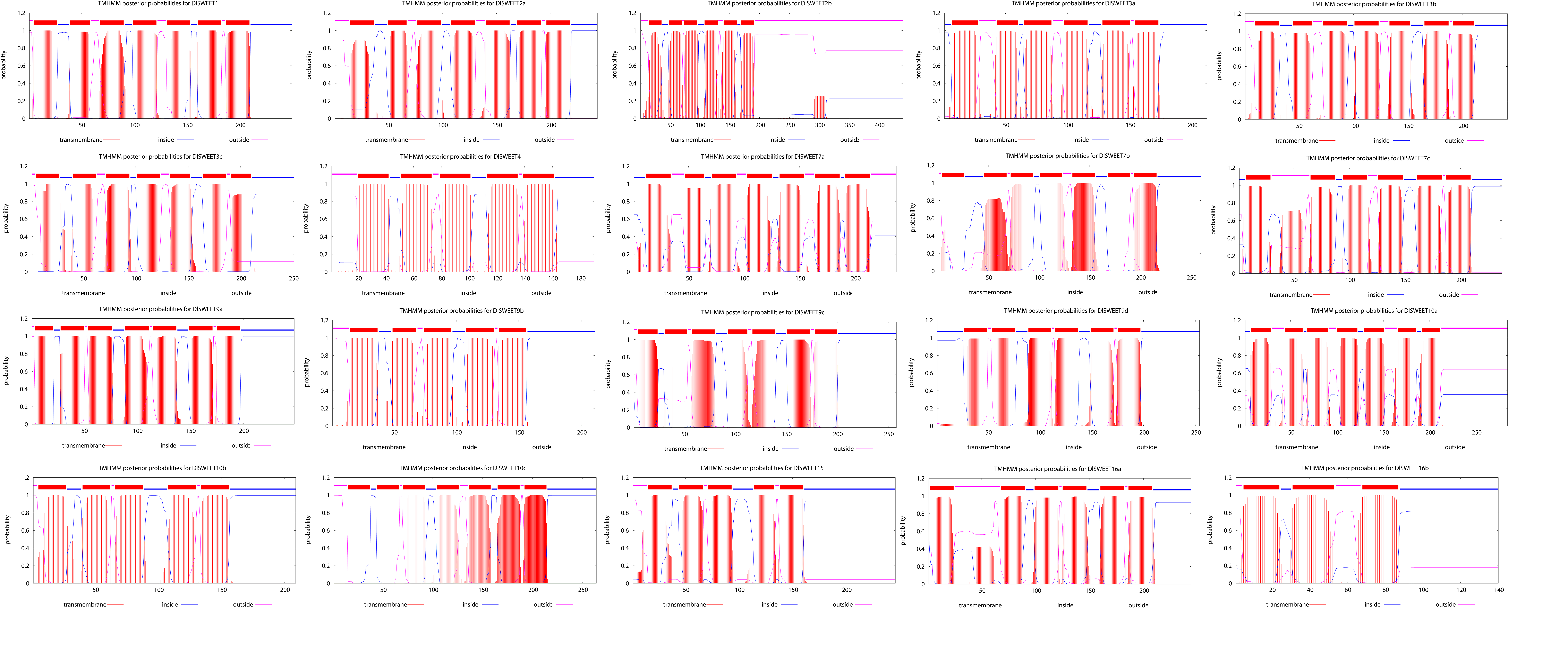

Supplement: Supplementary file 1 [file ijms-23-08914-s001.zip › Figure S1. The TMHs of DlSWEET proteins.tif]

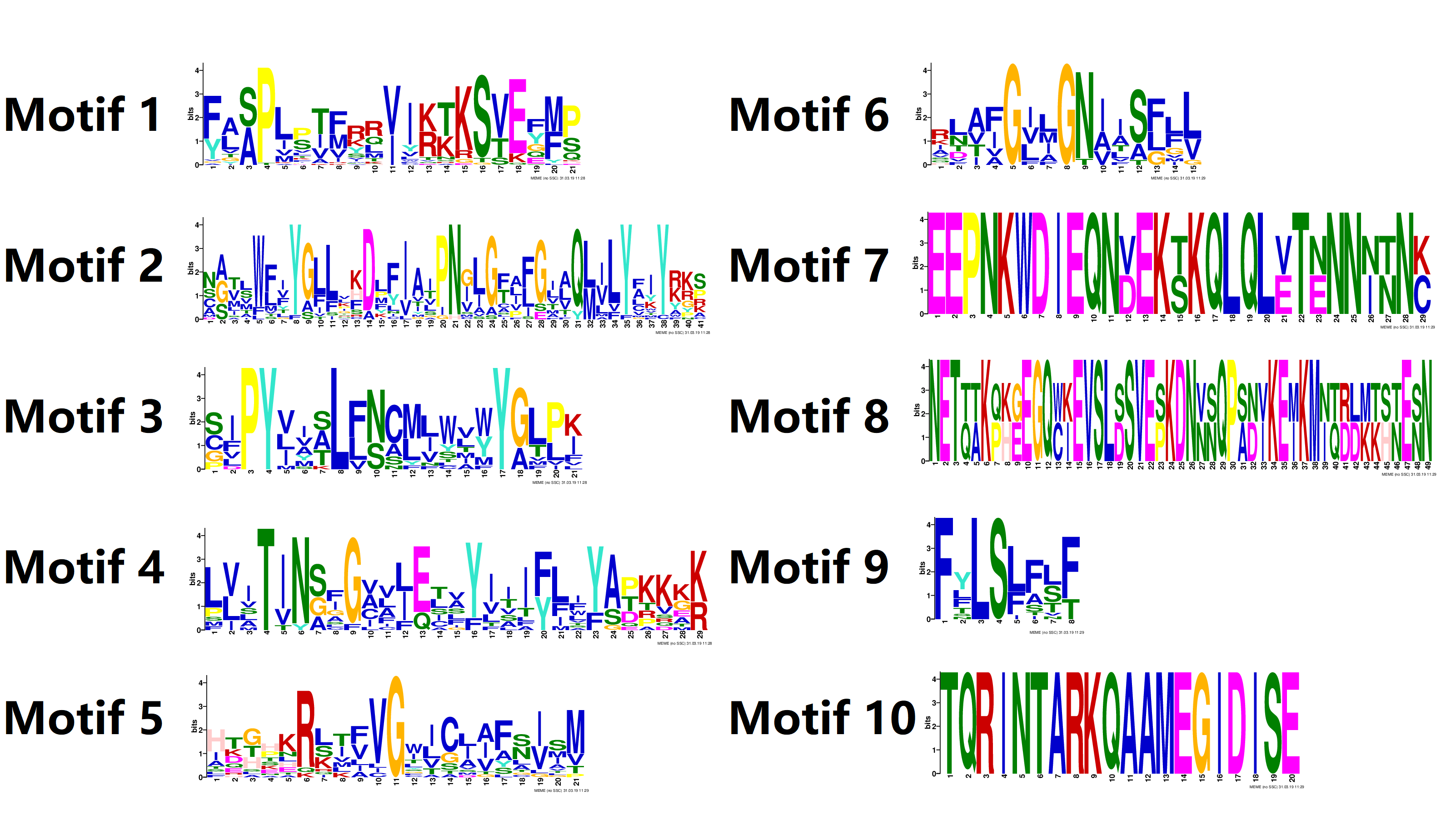

Supplement: Supplementary file 1 [file ijms-23-08914-s001.zip › Figure S2. The detailed information on each motif of DlSWEET proteins.png]
